# Un aspect très rare d'une grossesse extra utérine rompue

**DOI:** 10.11604/pamj.2015.21.12.6686

**Published:** 2015-05-06

**Authors:** Sarah Amourak, Mariam Tayae

**Affiliations:** 1 Service de GO II, CHU Hassan II, Fès, Maroc

**Keywords:** Grossesse extra utérine, rompue, sac gestationnel, Ectopic pregnancy, ruptured, gestational sac

## Image en medicine

C'est une patiente âgée de 37ans, primipare, ayant comme antécédent une stérilité secondaire de 17ans, admise aux urgences pour des métrorragies associées à des douleurs pelvienne sur une aménorrhée de 2mois. L'examen trouve une patiente tachycarde, TA à 90/50mmhg, un abdomen légèrement distendu, avec un saignement provenant de l'endocol. Echographie pelvienne objective un utérus de taille normal vide, présence en latéro utérin droit d'un sac gestationnel, avec un embryon dont la LCC correspond à 9SA, associé à un épanchement de grande abondance. Elle a bénéficié d'une mini laparotomie en urgence, l'exploration a montré un hémopritoine estimé à 1litre, un éclatement de la trompe et un sac gestationnel. Elle a bénéficié d'une salpingectomie droite. La Grossesse Extra-Utérine (GEU) est la nidation ectopique de l’œuf en-dehors de la cavité utérine. Cliniquement elle se manifeste par un retard de règles, des métrorragies classiquement noires, elles sont intermittentes, discrètes ou parfois plus abondantes, s'accompagnant de l’émission de caduque utérine et par des douleurs pelviennes, d'intensité variable, discrètes à syncopales L'examen clinique provoque une douleur à la palpation abdominale, avec parfois une défense sous-ombilicale. Les touchers pelviens notent un utérus moins gros que ne le voudrait l’âge gestationnel et peut percevoir une masse latéro-utérine douloureuse avec une douleur dans le cul-de-sac de Douglas. En règle, le toucher vaginal n'apporte que peu au diagnostic. Le pronostic vital dans les formes graves peut encore être en jeu. La GEU reste la première cause de mortalité au cours du premier trimestre de la grossesse, par hémorragie. Elle est l'exemple même de l'urgence chirurgicale. Le pronostic se pose plus souvent en termes de fertilité ultérieure chez les patientes ayant présenté une grossesse extra-utérine.

**Figure 1:**
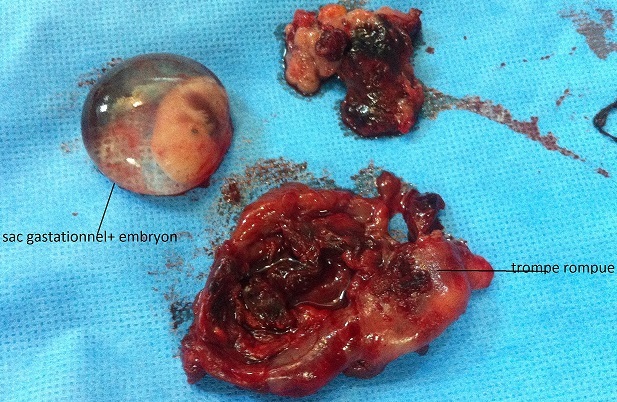
Sac gestationnel et trompe rompue

